# How do we improve men’s mental health via primary care? An evaluation of the Atlas Men’s Well-being Pilot Programme for stressed/distressed men

**DOI:** 10.1186/s12875-016-0410-6

**Published:** 2016-02-02

**Authors:** Anna Cheshire, David Peters, Damien Ridge

**Affiliations:** Department of Psychology, University of Westminster, 115 New Cavendish Street, London, W1W 6UW UK; Westminster Centre for Resilience, University of Westminster, 115 New Cavendish Street, London, W1W 6UW UK

**Keywords:** Mental health, Well-being, Male, Primary care, Stress, Masculinity

## Abstract

**Background:**

Over three-quarters of all suicides are men (England and Wales), this is despite higher levels of anxiety and depression being reported by women. This disparity may in part be explained by atypical presentations of distress in men, and gendered issues around help-seeking. Consequently, the Atlas Men’s Well-being Programme was designed to engage stressed/distressed men who were patients at a London-based GP surgery. Atlas encouraged GPs to identify and refer men for counselling and/or acupuncture by raising their awareness of men’s distress. The aim of this pilot study was to evaluate Atlas in terms of patients’ characteristics, service utilisation, patient outcomes and cost implications.

**Methods:**

All patients using the Programme were asked to complete a questionnaire before and after their Atlas sessions. Outcome measures included the Hospital Anxiety and Depression scale, Perceived Stress Scale, Warwick-Edinburgh Mental Well-being Scale, a 11-point scale measuring physical health, and the Psychological Outcome Profiles (PSYCHLOPS), a patient-generated outcome measure. Additionally, for cost calculations, participants were asked about their employment, number of days off work due to illness, and their health and social care service use.

**Results:**

102 participants were recruited, 82 completed pre- and post-treatment questionnaires. Comparisons pre- and post-treatment revealed a statistically significant improvement in anxious mood (*p* <0.001), perceived stress (*p* < 0.001), positive well-being (*p* = <0.001), PSYCHLOPS (*p* = <0.001) and physical health (*p* = 0.001), though not depressed mood (*p* = 0.660). Additionally, reductions in costs related to lost employment and health and social care use, exceeded the cost of Atlas counselling and acupuncture sessions, with an average saving of nearly £700 per patient.

**Conclusions:**

Atlas attendance was associated with improvements in patients’ mental and physical health, and demonstrated likely cost savings. It is now important to understand patient and stakeholder perspectives. Further research could compare usual care with the Atlas approach, and investigate full cost-effectiveness.

## Background

Men’s mental health is of growing concern in the UK. Recent figures show that in England and Wales 78 % of all suicides were male, and suicide is now the main cause of death in men aged 20–49 years [1–3]. Recent high profile male suicides - including celebrities from film and sport - have attracted the attention of media commentators, policy makers and practitioners [4–7], but questions of how best to address male mental health remain.

Although men are disproportionately more likely to take their own lives than women, fewer men than women are diagnosed with ‘common mental disorders’ [8], including depression [9–11] (although in low income populations the difference is much smaller). However, high levels of self-labelled “depression” are found in both men and women when they are asked in lay terms about depression [12], and men report work stress more frequently than women [13, 14]. Studies such as these suggest complex factors are influencing male distress, as well as the way that professionals interpret the issue. Further, there is under-reporting of male mental health problems: the ways in which men experience, express or indeed hide distress are poorly understood. A better grasp of these matters might enable distressed men to seek appropriate help sooner.

We define “distress” as a difficult emotional experience, which may be psychological, social or spiritual in nature (e.g. stress, anxiety, low mood). Distress can occur in a range of severities, which may or may not extend to a clinical diagnosis of mental health problems [15, 16]. The limited research on men and distress suggests similarities and differences between the genders in the ways they experience, express and manage their distress [17]. For example, both men and women can find it difficult to recognise and articulate their feelings [18], but men may be more likely to experience distress as anger directed at others [19], with under-utilisation of mental health services [20]. There is some evidence that men may want different things to women from their health professionals [18]. However, there are also variations between individuals that are not related to gender [18], and professionals may play a role in deterring men from help-seeking for distress [19, 21, 22]. Consequently, it is unhelpful to assume stereotypical or traditional masculine roles are the problem. Instead, mental health services oriented towards men need to be sensitive to the needs of diverse individuals [6, 17], present as ‘men-friendly’ [23], and be prepared to use a range of approaches [23].

People who are anxious or depressed are also known to access non-pharmacological interventions such as self-help, psychological treatments and complementary therapies [24–28]. Counselling is a psychological treatment, and umbrella term that covers a range of talking therapies that are delivered by trained practitioners to bring about effective change and improve well-being, allowing people to talk about feelings and problems in a confidential and dependable environment [29, 30]. Systematic reviews have shown that for mental health and psychosocial problems, counselling is more effective than usual care in the short term; is suitable for heterogeneous primary care populations; is as effective as cognitive behavioural therapy (CBT); evokes high patient satisfaction; and the costs are similar to usual care [31–34]. Additionally, private acupuncturists commonly treat stress, anxiety and depression [35], and there is good evidence that acupuncture can play an effective part in treating certain stress-related problems, anxiety and depression [36–39]. Western science is still to fully establish how acupuncture works. However, neurophysiological mechanisms are thought to be important, for example, the insertion of needles can release muscle tension, override unhelpful brain signals and affect endorphins [40, 41]. In addition, non-specific effects are thought to play an important role in the effectiveness of treatment. Non-specific effects include the therapeutic relationship with the acupuncture practitioner (talking and being listened to), and the promotion of self-awareness, self-confidence and self-responsibility as a central concept within Chinese Medicine [42, 43].

It was felt that counselling could provide an effective talking-based intervention for men as an alternative to CBT, which is already available on the NHS. Counselling was considered suitable for delivering the safe environment we know men need to be able to open up [44]. Acupuncture was considered a way to initially engage ‘stressed’ men in a treatment that is not predicated on talking about difficult emotions. This ostensibly ‘non-psychological’ entry point, while providing professional support and treatment, also allows psychological options and the relevance of referral to counselling to be explored [45]. In addition, because distressed men commonly experience somatic symptoms such as pain, insomnia, and digestive problems [46, 47], there might be advantages in offering a physical therapy option [48]. Whilst another body centred therapy (e.g. massage) may also have been appropriate, acupuncture was selected as it has a strong professional body; there is research to suggest it has an impact on anxiety and bodily discomfort both of which are common ways that men present their distress; and the Atlas Team has successful experience of providing acupuncture on the NHS [49, 50].

Primary care services play an important role in the identification and encouragement of men with mental health problems to get help [6]. Although counselling and acupuncture can be helpful for treating distress, the specific effectiveness (i.e. how well interventions work in the real world [51]) of providing these services for men in primary care is unknown. The Atlas Men's Well-being Programme was a pilot service that aimed to implement what we already know about counselling, acupuncture and engaging men in distress, and to work with GPs to develop a ‘male sensitive’, primary-care-based service that would be acceptable both to GPs and men presenting with stress or distress. This paper reports on a service evaluation of the Atlas pilot in terms of patient characteristics, service utilisation, patient outcomes and cost implications.

## Methods

### Ethics, consent and permissions

Ethics approval for the evaluation was obtained from the University of Westminster Ethics Committee (reference: 12-13-07). An application was made to NRES Queries, who confirmed on 17 July 2012 (ref 04/26/50) that NHS ethical approval was not required due to the service evaluation nature of the study. Informed written consent was collected from all participants.

### Participants

**Fig. 1:**
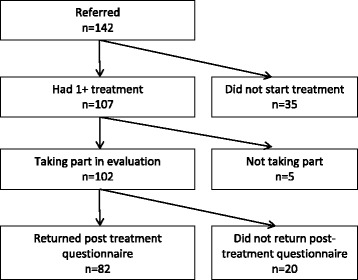
Flow of participants through the Atlas Men's Well-being Programme evaluation

All patients referred to Atlas were invited to take part in the evaluation. Referrals were made to the Programme by Victoria medical centre (VMC) GPs, who referred patients attending routine GP appointments and presenting with stress/distress. Referral decisions were based on guidelines provided to GPs by the Programme Director, taking patient treatment preference (Atlas counselling, Atlas acupuncture or a non-Atlas option such as IAPTs) into account. Thus, because referrals were based on conversations between GPs and patients, we are unable to know the number of patients where referral to Atlas was discussed but another option chosen. We did ask GPs about their experiences with Atlas, but GPs did not indicate that men declining the Atlas option was an issue. Inclusion criteria included: male; registered patient at the VMC/VMC branch; aged 18 years or over; and suffering from mild to moderate stress or distress – which was judged by the referring GP, but attempted to take into account the less usual ways men express distress e.g. lack of emotional vocabulary, somatic symptom presentations. Exclusion criteria included: current substance abuse issues and serious mental health problems. Presence of serious mental health problems was also judged by the referring GP. In addition, if a participant returned extremely high anxiety and depression scores on their questionnaire or the Atlas practitioner was alerted to the presences of serious mental health problems during the initial consultation it was protocol to inform the referring GP and Atlas service director. One-hundred and forty-two patients were referred to the Programme, of these 35 (25.3 %) did not attend any appointments and five did not want to participate in the evaluation. Therefore 102 patients participated in the evaluation (see Fig. 1).

### The intervention: the Atlas Men’s Well-being Programme

The Atlas Men’s Well-being Programme was based at the Victoria Medical Centre (VMC), a large GP practice in central London which serves a broad demographic of patients. Atlas is a Greek God who carried the world on his shoulders, this name was chosen for the programme as a representation of men’s struggles. The Service operated from March 2013 to July 2014. The Programme was initially conceptualised by Ridge (the Principle Investigator) with the current literature on men, gender and distress in mind. Then, in consultation with Peters (the Service Director), the pilot was co-designed with input from VMC staff and an Expert Advisory Panel, including a male patient at the practice. Atlas offered counselling and acupuncture to men who were patients at the VMC. Appointment making was integrated into the practice’s computer-based system, so that patients could book their sessions in the normal way via the practice reception. Decisions about patients’ treatments were delegated to the practitioners who were free to treat as they would in real life. Patients could have up to 12 sessions of counselling and/or 6 sessions of acupuncture. Due to the subsequent popularity of Atlas, this schedule was altered six months after initiation to be up to 12 sessions in total, rather than the original 18. However, in exceptional circumstances, there was the option for patients to obtain additional counselling/acupuncture if agreed by the patient, practitioner and Atlas Service Director.

#### Counselling

Patients referred to counselling received weekly, 1-hour sessions of integrative/humanistic counselling [52]. Sessions were delivered by experienced counsellors (two male, one female, five to seven years post-qualification experience) who were selected for their high levels of experience in working with men and their male friendly approach (as demonstrated to a panel of experts e.g. skilled at helping with the sorts of mental health issues men are prone to such as anger and its management, work and relationship problems). Counsellors were all registered with professional bodies (The UK Council for Psychotherapy and/or The British Association for Counselling and Psychotherapy).

#### Acupuncture

Patients referred to acupuncture received weekly sessions (lasting 30 min) of individualised Traditional Chinese Medicine acupuncture treatment. Acupuncture sessions were delivered by senior acupuncturists (one male, one female, 11 to 17 years post-qualification experience), both of whom had experience of working in the NHS and with men, and were registered with the British Acupuncture Council. During the first session a full case history was taken along with traditional pulse and tongue diagnosis. From these, a treatment plan was developed, which could be adjusted each week depending on the patient’s response to treatment.

#### Male-sensitive

Designing the service was based on what current research already tells us and on a guidelines for making mental health services male-sensitive [53]. For example, guidelines suggest: start by understanding the obstacles to men seeking help and using services, which may be specific to a particular community of men; communicate with men in a way that respects their maleness to help minimise the stigma many men feel, because of the threat psychological distress can pose to their sense of themselves *as men*; and plan evaluation from the outset and make the results widely known to improve the evidence base and spread good practice. In practical terms this included: providing two different options (counselling/acupuncture) that men could use to approach their problems, as research has shown that a one size fits all approach does not work for men; recruiting both male and female practitioners who were positive about men, experienced in working with them, and able to demonstrate an understanding and approach sensitive to the needs of men; using advertising material deemed appealing to a male focus group in look and content; gaining stakeholders’ advice on the language which should be used in a mental health service for men e.g. use of term ‘well-being’ as opposed to ‘mental health’; making the service male only; and briefing GPs about typical presentations of male distress e.g. somatic symptoms, lack of emotional vocabulary.

### Outcomes

Patient outcome, cost, and service use data were collected using pre- and post- treatment patient questionnaires, using consecutive sampling. Service use data were also collected from electronic patient records. The following data were collected from patients:

The Hospital *Anxiety and Depression* Scale (HADS) [54] was the primary outcome measure. It is a validated and widely used measure comprising anxiety and depression subscales containing seven items. Each item is scored on a scale of 0 to 3, therefore scores range from 0 to 21. The scale has established validity and reliability [55].

The *Perceived Stress* Scale (PSS) scores reflect the level of stress experienced by the respondent and not psychological symptomology [56]. The 10 PSS items are scored on a scale of 0 to 4, and summed to give a total score between 0 and 40. Its validity and reliability are well-established [56].

The *Warwick-Edinburgh Mental Well-being* Scale (WEMWBS) [57, 58] measures positive well-being. The seven items have five response categories which are scored from 1 to 5, providing a total score ranging from 7 to 35. The scale has established validity and reliability [57].

The *Psychological Outcome Profiles* (PSYCHLOPS) [59] was developed to assess patient-centred mental health outcomes in primary care-based talking therapy. Patients select and rate their two main ‘problems’ and rate their severity from 0–6. Patients then rate their functioning and well-being on similar 6-point scales. Items are summed to provide a total score ranging from 0 to 20. An additional question on the PSYCHLOPS post-treatment questionnaire asks ‘Compared to when you started your sessions, how do you feel now?’ The scale - approved by the Plain English Campaign - has been shown to be valid and reliable [60, 61].

*Physical health* was measured using an 11 point Likert scale anchored 0 (extremely poor) to 10 (Excellent).

In order to estimate *cost implications*, Atlas patients were asked about their *employment status, number of days off work due to illness,* and *health and social care service use* over the preceding 12 weeks.

On the pre-treatment questionnaire only d*emographic data* (age, ethnicity) were collected, and patients were asked how they had *first heard about Atlas* and if they were using *anything to help deal with their stress (i.e. prescription medication, over the counter remedies, alcohol, other)*.

#### Procedure

On referral to Atlas by their GP, patients were asked to go to reception and pick up an evaluation pack and book their Atlas appointments. Evaluation packs comprised a covering letter, patient information sheet, consent form, pre-questionnaire and stamped addressed envelope for returning the questionnaire to the Evaluation Team. Patients could return their completed pre-treatment questionnaire and consent form to the Evaluation Team prior to their first session, either through the post or via VMC reception (there was a box behind reception where questionnaires could be left for the researcher to pick up). Patients were sent their post-treatment questionnaire by the researcher once they had finished using Atlas (i.e. completed all their counselling/acupuncture appointments or withdrawn). Identical copies of the questionnaires were also available to be completed online, according to patient preference. Questionnaires did not use patient names, rather date of birth and initials for identification.

#### Data analysis

Quantitative data were analysed using SPSS version 19. To ensure a conservative analysis, non-parametric tests (Mann Whitney-U, Wilcoxon Signed Rank, Kruskal-Wallis, McNemar and Chi-square as appropriate) were used throughout. Initially, data were examined for differences between those who did and did not return their post-treatment questionnaire, for baseline and demographic variables. A Bonferroni adjusted alpha level of 0.005 (0.05/9) was used. To examine patient outcomes Wilcoxon Signed Rank tests were used to compare pre- and post-treatment data including the HADS, PSS, PSYCHLOPS, WEMWBS and physical health. A Bonferroni adjusted alpha level of 0.01 (0.05/5) was used. Level of HADS anxiety and depression were calculated using recommended cut-off scores: No anxiety/depression (0–7), low anxiety/depression (8–10), moderate anxiety/depression (11–15), severe anxiety/depression (16–21) [62]. Magnitude of effect sizes (r) were established using the Cohen criteria for r [63] of 0.1 = small effect, 0.3 = medium effect and 0.5 = large effect. In addition, a Kruskal-Wallis test was used to compare patients by treatment received (counselling, acupuncture or both), in order to establish if different treatment options had an effect on patient outcomes. The p-value was set at 0.05 for this exploratory test.

For the cost implications, the costs of service use and the cost of lost employment were estimated for the 12 week period before the baseline visit and the 12 weeks prior to follow-up (data were collected on the pre- and post-treatment questionnaire). Public sector unit costs for services were obtained from Department of Health sources [64, 65]. Private sector unit costs were estimated from web searches. Unit costs were multiplied by the number of hours of use of each service to estimate the total costs. Based on estimates of annual salaries, the cost of lost employment was calculated.

## Results

### Participant characteristics (*n* = 102)

The spread of ages attending Atlas, though wide, was skewed towards younger and middle age men. The range of ethnicities was similar to the profile of patients registered at VMC (Table 1). Eighteen percent of participants were unemployed. Two-thirds of participants were taking medication to help deal with their stress/distress, most frequently prescription medicines (37.3 %) and over the counter remedies (24.5 %). Nearly one fifth (18.6 %) said they were using alcohol to help them cope with stress or distress.

**Table 1:** Participant statistics

Age	median 41.0 years old, interquartile range 31–49	
Employment^a^		
Paid or self-employed		74.5 %
Voluntary work		2.0 %
Unemployed		17.6 %
Student		6.9 %
Retired		3.9 %
Exempt through disability		3.9 %
Other		3.9 %
Ethnicity		
	VMC patients	Atlas patients
White – British	44.3 %	48.0 %
White – European/other	29.0 %	26.5 %
Black/Caribbean/African	6.9 %	7.9 %
Asian	10.8 %	3.9 %
Arabic	0.8 %	3.9 %
Mixed	3.0 %	3.9 %
Other	5.3 %	3.9 %
Missing	-	2.0 %

^a^participants were able to indicate more than one employment category

**Fig. 2:**
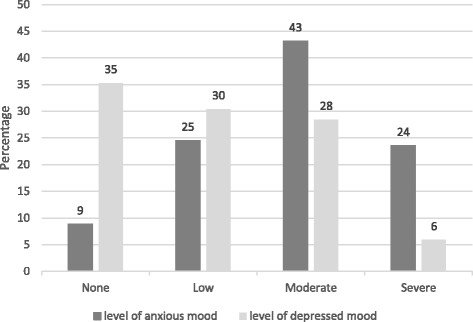
Level of anxiety and depression in patients prior to commencing Atlas sessions, by percentage

Although HADS data showed wide variation in levels of anxious and depressed mood, anxious mood was more prevalent in this population than depressed mood (see Fig. 2).

### The Atlas Programme service use (*n* = 102)

Most patients had initially heard about the Programme through their GP (88.2 %), rather than only through the Atlas publicity made available throughout the surgery - leaflets (4.9 %), posters (1.0 %). However, GPs frequently provided leaflets to men on referral, making men aware of Atlas as a service.

Both counselling and acupuncture treatments were popular, but counselling was accessed most (see Table 2). Patients receiving counselling had an average of 8.0 sessions (standard deviation: 4.5; range: 1 to 23). Those receiving acupuncture attended an average of 5.5 sessions (standard deviation: 2.0; range: 1 to 11). As there were no statistically significant differences between treatment received (counselling, acupuncture or both) and demographic variables and baseline psychosocial well-being scores, it seems that patient demographics and levels of distress were unrelated to choice of treatment.

**Table 2:** Treatments received by Atlas patients

Treatment received	Number	Percentage
Counselling only	39	38.2
Acupuncture only	26	25.5
Counselling and acupuncture	37	36.3

**Fig. 3:**
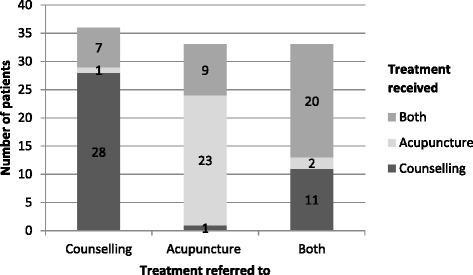
Treatment routes taken through the Atlas programme

We examined the routes Atlas patients took through the Programme. If an Atlas practitioner felt a patient would benefit from another Atlas modality (as well as or instead of their own) they could cross-refer. Most patients attended for the treatment they were referred to (73.1 %) but around a quarter had a different treatment. 15.9 % were cross-referrals between Atlas practitioners. 9.7 % were referred for both treatments but only attended one – nearly always counselling (Fig. 3).

### Patient outcomes and cost implications

Of the 102 patients who completed their Atlas pre-treatment questionnaire, 82 (80.4 %) also completed their post-treatment questionnaire (Fig. 1). Statistically significant differences were found between responders and non-responders on age (*p* = 0.002): those who did not complete their post-treatment questionnaire were younger. All statistical analyses were based on the 82 completed data sets.

#### Changes in anxious and depressed mood

Comparisons between pre- and post-treatment revealed statistically significant improvements in anxious mood (p <0.001), with a medium effect size (*r* = 0.38). There was no change in depressed mood (*p* = 0.660), see Table 3.

**Table 3:** Study variable scores over time pre- and post-treatment

	Pre-treatment		Post-treatment		*p*-value*	Effect size (r)
	Median score (interquartile range)		Median score (interquartile range)			
HADS – anxious mood (range 0–21 higher score = worse)	13.0	(9.0–15.3)	9.0	(8.0–12.0)	<0.001	0.38
HADS – depressed mood (range 0–21 higher score = worse)	9.0	(6.0–11.0)	9.0	(6.0–11.0)	0.660	0.03
PSS (range 0–40 higher score = worse)	24.0	(21.0–28.3)	19.0	(12.0–24.0)	<0.001	0.36
PSYCHLOPS (range 0–20 higher score = worse)	16.0	(14.0–17.0)	8.0	(5.0–13.0)	<0.001	0.57
WEMWBS (range 7–35 higher score = better)	20.0	(16.0–23.0)	25.0	(20.0–28.0)	<0.001	0.40
Physical health (range 0–10 higher score = better)	7.0	(5.0–7.0)	7.0	(5.0–8.0)	0.001	0.25

*Calculated using a Wilcoxon Signed Rank test

*HADS* hospital anxiety and depression scale, *PSS* perceived stress scale, *PSYCHLOPS* psychological outcome profiles, *WEMWBS* Warwick and Edinburgh well-being scale

Over a third of patients did not have depression (classified using recommended cut-off scores for the HADS [62], see Fig. 2). A sub-analysis was conducted comparing levels of pre- and post-treatment depressed mood, excluding patients with no depression from the analysis. Comparisons of patients with depressed mood (low, medium or high levels of depressed mood, *n* = 50) revealed a statistically significant improvement in depressed mood (p <0.001).

#### Changes in other study variables

Comparisons between pre- and post-treatment revealed a statistically significant improvement in perceived stress (*p* < 0.001) with a medium effect size (*r* = 0.36); PSYCHLOPS (*p* = <0.001) with a large effect size (*r* = 0.57); positive well-being (*p* = <0.001) with a medium effect size (*r* = 0.40); and physical health (*p* = 0.001) with a medium effect size (*r* = 0.38), see Table 3.

The majority of men (78.0 %), who were asked ‘Compared to when you started your sessions, how do you feel now?’ said they felt better than they had prior to starting Atlas. Eleven (13.4 %) said they did not feel much different and three (3.7 %) felt a little worse. Four (4.9 %) did not answer this question.

Patients were compared by treatment received (counselling, acupuncture or both), in order to establish if different treatment options had an effect on patient outcomes. Results showed that change in perceived stress (*p* = 0.030) and PSYCHLOPS (*p* = 0.049) scores were affected by patient group. That is, patients receiving counselling only improved more than patients who received acupuncture only. There were was no additional improvements among patients receiving both treatments.

Cost calculations point to reduced costs related to health and social care utilization and lost employment compared with the 12 weeks prior to starting Atlas sessions. These reductions in costs exceed the cost of the Atlas counselling and acupuncture sessions themselves, with an average of saving of nearly £700 per person (Table 4). The standard deviation values related to this data are very large because, as is typical with cost data, they are highly skewed with a long right hand tail [66].

**Table 4:** Summary of costs (in £) related to Atlas per patient

	Pre	Post
	Mean (SD)	Mean (SD)
Counselling/Acupuncture sessions cost	0	661 (371)
		[*n* = 102]
Health and social care costs	658 (3,675)	92 (194)
	[*n* = 102]	[*n* = 82]
Lost employment	891 (2,574)	53 (200)
	[*n* = 102]	[*n* = 82]
Total	1,548 (4,411)	855 (504)
	[*n* = 102]	[*n* = 82]

## Discussion

The recent increase in male suicides and the widening suicide gender gap have highlighted the importance of working with men to support their mental wellbeing. We conducted an evaluation of the Atlas Men’s Well-being Programme – a ‘male-friendly’ pilot programme based in primary care, designed to support men suffering from stress and distress. We examined patient characteristics, service use, outcomes, and the cost implications of the Atlas service. Our findings suggest that a service provided in this way can engage a diverse sample of men and provide positive outcomes for well-being, while also being cost-effective.

Atlas was marketed to health professionals and patients as a service for stressed/distressed men. Indeed, men reported reductions in stress and anxiety after using Atlas. In addition, among the men who reported problems with depression before using Atlas, there was a statistically significant reduction in depression. Changes reported in physical health suggest Atlas could address the needs of men who experience somatic symptoms in association with stress/distress. The largest improvement however, was found for the PSYCHLOPS, an outcome measure which allows participants to select and rate the problems bothering them most. This finding suggests that when evaluating a service such as Atlas, where patients attend for a variety of reasons, using a patient-centred outcome to identify change is particularly important.

A key finding of this evaluation was the important role that GPs played in identifying and encouraging men to use Atlas (most men had first heard about Atlas through their GP). This supports findings published by the Men’s Health Forum and MIND, which suggest that primary care has a key role to play in the identification and support of men with mental health problems [6]. However, it can be challenging for GPs to recognise mental health problems because their patients may be reluctant to acknowledge (to their GP or even themselves) that they are suffering from emotional distress [67]. In addition, a common presentation of symptoms for depression for men may not be mood disturbance or psychosocial symptoms, but somatic symptoms (such as fatigue and pain), sleep problems and anger [46]. This requires GPs to be skilled in looking beyond the physical problems presented to recognise anxiety and depression [6, 67]. GPs referring to Atlas for this study had experience of working with an in-house counsellor prior to Atlas. They were also involved in the Atlas set-up period, which included ongoing discussions around identifying male distress, thus promoting GP skills in this area. Both GP training to identify men with mental health problems *and* appropriate resources to provide subsequent treatment need to be in place [67]. The consequences of getting this right is that a wide demographic of men can be reached, including hard to reach groups who might be at greatest risk, such as ethnic minorities (23.5 % BME in Atlas) and younger/middle aged men.

Both counselling and acupuncture treatments were well utilised with a range of treatment options (i.e. counselling, acupuncture or both) chosen, suggesting that men adopted varying treatment preferences to address their problems. This supports findings that flexibility needs to be built into services for men (e.g. talking versus manual therapies) [6, 17]. However, the evaluation challenged the stereotype that ‘men do not talk’, as counselling was in fact more often used than acupuncture. It seems then that men are willing to talk when the setting (which includes affirmative messages about men seeking mental health support) is right. This supports research that men can be willing to seek help [68] and will talk when safe to do so [69, 70]. The fact that current trends suggest an increase in men accessing talking therapies may signal that gender relations are changing for the better [71, 72].

The service use figures also point to a willingness amongst GPs to refer to both counselling and acupuncture. GPs can sometimes be reluctant to refer their patients to a complementary therapy due to their own lack of knowledge; a perceived lack of evidence to support complementary therapy use; or the legal implications of promoting complementary therapy use [73–75]. However, acupuncture may be more acceptable to GPs: it is one of the most common complementary therapies that GPs in the UK refer their patients to [73, 76], and many GPs feel that it should be more widely available on the NHS [77]. GP referral to talking therapies, like counselling, is much more established since the introduction of IAPTs in 2011. However, there is evidence to suggest that direct contact with counsellors is likely to increase GP referral to the service [78], suggesting an in-house service, such as Atlas, supports GP referral.

Oliffe and colleagues [23] have highlighted that male-friendly services are best delivered through ‘diverse modes and methods’. Atlas represents one such adaptation, but other types of male-friendly services should be evaluated so that the pros and cons of different approaches can be better understood. For example, it is not clear whether it is most beneficial for a service to be ‘male-only’ or if a mixed gender service marketed for men could achieve similar results. Additionally, interventions such as Atlas can only be viewed as part of the solution to the challenges in men’s mental health. Interventions need to form part of a wider strategy to understand distress in men and support men to seek help. The charity CALM (Campaign Against Living Miserably) suggests that our society’s expectations of, and assumptions about men; the ways they are portrayed in the media; public service provision; role in the workplace and family life will all have to be re-thought [79]. Researchers and charities like CALM have also argued that services for men will need to go where men commonly gather, e.g. sports clubs, the Internet [23, 80].

Our service evaluation was a pilot, single-site programme, and its limitations centre around the generalisability of findings to other populations (e.g. rural GPs practices). As there was no control group, further research is needed to confirm that improvements were due to treatment. On the other hand, many of the problems that men bought into Atlas (e.g. deep and long-standing issues and problems and the desire to talk them through) were unlikely to be remedied on their own. In addition, the cost calculations only included a limited number of variables (lost employment, service use and cost of practitioners’ time), management and indirect overheads are not included, nor are the wider benefits of improving health and well-being that Social Return on Investment analysis, or a thorough cost analysis, may reveal [81]. There are limitations of estimating income for individuals within surveys whether using self-reported data or relying on internet searches. Internet search was the selected method because self-reported income data tends to have high non-response and/or is miss-reported by a significant subset of respondents [82, 83]. There were some differences in outcome found depending on the type of treatment received. However, this sub-group analysis comprised small groups, thus a larger sample would be required to confirm these difference further. Additionally, qualitative data could add to our understanding of the benefits and challenges of delivering a programme like Atlas.

## Conclusions

Our findings suggest that a service provided in this way, can engage men of widely diverging age, ethnicity and class, with positive outcomes for men’s well-being, while saving costs to society. These findings provide the foundation on which to build future, larger research studies, which could include multiple sites and a control group. A larger sample would also allow for comparisons between treatment received (counselling, acupuncture or both) and usual care, allowing further understanding of the benefits of the different modalities and when to use each treatment. Findings suggest that outcome measures used in this study would be appropriate for a larger trial. In addition, findings point to potential cost savings related to using Atlas, suggesting a more comprehensive cost-analysis study could reveal the potential savings to the NHS and society more widely.

## Abbreviations

BME: black and minority ethnic
CALM: campaign against living miserably
CBT: cognitive behavioural therapy
HADS: hospital anxiety and depression scale
NHS: National Health Service
PSS: perceived stress scale
PSYCHLOPS: psychological outcome profiles
VMC: Victoria medical centre
WEMWBS: Warwick and Edinburgh mental well-being scale
IAPT: increasing access to psychological therapies
